# Hypomorphic RAG2 Deficiency Promotes Selection of Self-Reactive B Cells

**DOI:** 10.1007/s10875-024-01849-9

**Published:** 2025-01-15

**Authors:** Christopher D. Thouvenel, Christopher M. Tipton, Yasuhiro Yamazaki, Ting-ting Zhang, Stacey Rylaarsdam, Jennifer R. Hom, Catherine Snead, Chengsong Zhu, Quan-Zhen Li, Yu Nee Lee, Tomoki Kawai, Neshatul Haque, Michael T. Zimmermann, Sivasankaran Munusamy Ponnan, Shaun W. Jackson, Rich G. James, Ignacio Sanz, Luigi D. Notarangelo, Troy R. Torgerson, Hans D. Ochs, David J. Rawlings, Eric J. Allenspach

**Affiliations:** 1 Center for Immunity and Immunotherapies, Seattle Children’s Research Institute, Seattle, WA USA; 2 Lowance Center for Human Immunology, Emory University School of Medicine, Atlanta, GA USA; 3 Laboratory of Clinical Immunology and Microbiology, National Institute of Allergy and Infectious Diseases, National Institutes of Health, Bethesda, MD USA; 4 Immunome Inc, Bothell, WA USA; 5 Department of Immunology, Microarray and Immune Phenotyping Core, University of Texas Southwestern Medical Center, Dallas, TX USA; 6 Genecopia Inc, Rockville, MD USA; 7 Pediatric Department A and the Immunology Service, Ramat-Gan and Sackler Faculty of Medicine, “Edmond and Lily Safra” Children’s Hospital, Jeffrey Modell Foundation Center, Sheba Medical Center, Tel Hashomer, Tel-Aviv University, Tel-Aviv, Israel; 8 Bioinformatics Research and Development Laboratory, Linda T. and John A. Mellowes Center for Genomic Sciences and Precision Medicine, Medical College of Wisconsin, Milwaukee, WI USA; 9 Department of Pediatrics, University of Washington, Seattle, WA USA; 10 Allen Institute for Immunology, Seattle, WA USA; 11 Department of Immunology, University of Washington, Seattle, WA USA

**Keywords:** Hypomorphic RAG deficiency, VH4-34, Hyper-IgM, Marginal zone B cell

## Abstract

Reduced function or hypomorphic variants in recombination-activating genes (RAG) 1 or 2 result in a broad clinical phenotype including common variable immunodeficiency (CVID) and even adult-onset disease. Milder RAG variants are less characterized. Here we describe the longitudinal course of a milder combined RAG deficiency in 3 of 7 siblings sharing the same RAG2 mutations over a 50-year study. Whole-genome and repertoire sequencing, bacteriophage immunizations, and deep immunophenotyping were used to compare affected and unaffected family members. The clinical phenotype of three affected siblings with hypomorphic RAG deficiency ranged from combined immunodeficiency and early mortality to a late-onset CID with hyper-IgM phenotype. T cells were remarkably similar across affected siblings, yet CDR3 skewing and regulatory T cell defects were not observed. B cell analysis showed elevated unswitched CD27+ and CD21^low^ cells as well as features of an autoreactive antibody repertoire and presence of secreted autoantibodies, yet no clinical autoimmunity was present. Most striking was an expanded polyclonal marginal zone-like B cell population (IgM+IgD+CD27+) utilizing the self-reactive unmutated VH4-34 receptor demonstrating that hypomorphic RAG deficiency can promote expansion of self-reactive B cells. This process, however, was not sufficient to trigger clinical autoimmunity. Utilizing multiple approaches, we functionally measured the specific RAG2 variant effects and assessed how selection and secondary triggers altered the BCR repertoire and immunophenotype overtime. Overall, we demonstrate a broad disease spectrum in siblings with identical hypomorphic RAG deficiency, highlighting that phenotypic divergence can result from expansion of IgM + memory B cells.

**Supplementary Information:**

The online version contains supplementary material available at 10.1007/s10875-024-01849-9.

## Introduction

*RAG1* and *RAG2* genes are essential for V(D)J recombination and lymphocyte development [1]. Biallelic hypomorphic *RAG* variants that partially decrease the function of the RAG complex have been associated with a spectrum of diseases ranging from combined immunodeficiency to milder immune defects [2]. Cases of adult-onset biallelic RAG deficiency have been reported and are likely underdiagnosed [3–5]. Severe RAG variants often result in early onset and highly penetrant disease, yet milder recombination defects are frequently associated with presentations later in life, often elicited by environmental triggers. Although disease severity mainly correlates with residual recombination activity [6, 7], rare siblings and cohorts with identical RAG variants have demonstrated divergent presentations suggesting recombination activity is not the only phenotypic driver [8–17]. RAG deficiency is often studied in aggregated cohorts of patients with various *RAG1* or *RAG2* mutations that includes a range of recombination efficiencies [2, 18–20]. Most studies have focused on severe RAG variants, while milder variants are harder to classify [21]. Detailed family studies comparing multiple affected individuals with less penetrant hypomorphic RAG variants are thus needed to further understand immune dysregulation phenotypes.

RAG deficiency has been consistently associated with dysregulated lymphocyte subsets [19]. Restricted pre-immune T cell repertoires and T cell lymphopenia have been observed in hypomorphic RAG deficiency along with altered thymic selection and regulatory T cell defects [22]. In contrast, the B cell compartment can show more variability [19]. An outstanding question remains whether these divergent B cell phenotypes reflect genetic background, selection and/or environmental exposures. Recently, a cohort of subjects with hypomorphic RAG deficiency was described across a diverse set of RAG1/2 variants. While transitional and resting naïve B cells were found reduced, expansions in CD21^low^T-bet^+^ B cells and non-switched (IgM+IgD+CD27+) B cells were observed [20, 23]. Peripheral blood IgM+IgD+CD27+ B cells are a heterogenous population that includes both unswitched memory B cells and IgM^hi^ marginal zone (MZ)-like B cells [24]. The MZ-like cells in peripheral blood are named for a shared cell surface phenotype with splenic MZ B cells defined by microanatomy and exclusion from the follicular zone. MZ B cell receptors are enriched in poly-/self-reactivity yet are not typically pathogenic and secrete natural IgM in the steady state [25]. Hyper IgM-like phenotypes have been observed in RAG deficiency, but the B cells were not analyzed [12, 26]. Further characterization of the IgM + B cells in hypomorphic RAG deficiency is needed.

In this study, we describe an affected family where 3 of 7 siblings presented with hypomorphic compound heterozygous variants in the *RAG2* gene. We conducted a comprehensive analysis that included clinical and immunologic data, whole-genome sequencing, and deep T and B cell repertoire data compared over five decades. This analysis also encompassed serum analysis and evaluation of self-reactive receptors, such as the VH4-34 expressing B cell clonotypes. We compared affected siblings with hypomorphic RAG deficiency versus their healthy siblings or public datasets to generate a unique longitudinal description of immune phenotype across siblings in the setting of a milder recombination defect.

## Methods

### Human Subjects

All individual participants gave their written informed consent under the Immunology Biorepository #11970 and/or #667 protocols approved by Seattle Children’s Hospital Institutional Review board. For the analysis of the recombination activity of the RAG2 variants, patient consent was obtained according to protocol 18-I-0041 (NCT03394053) approved by the National Institutes of Health IRB. Bacteriophage immunization was described previously [27] and timed blood samples were collected by standard phlebotomy. Autopsy was performed according to standard practices.

### Genomic Sequencing and Protein Modeling

RAG2 variants were identified via whole genome sequencing as described previously [28] and confirmed via Sanger sequencing. PBMC or FFPE autopsy material (QIAamp DNA FFPE tissue Kit; QIAGEN, Germany) was used to confirm genotypes via Sanger sequencing. The core domain of human RAG2 (residue 1–350 aa) based on the mouse RAG1/2 core complex CryoEM model (PDB ID 6oem and resolution 3.7 Å) was used for modeling. The C-terminal domain (Plant Homeodomain, PHD, residue 410–480 aa) model and high-resolution mouse template (PDB ID 2V88 and resolution 2.0 Å) were leveraged for a two domain-based model. 3D variant models of G243V and C423Y were then created using the computer program FoldX v5.0 followed by their energetics calculation and electrostatic visualization using APBS and PyMOL.

### PBMC Analysis and Culturing

PBMC were isolated and processed for multicolor flow cytometry using standard protocols. Data were acquired on a FACS LSRII (Becton Dickinson; USA) and/or sorted with FACSAria II (BD Bioscience) and evaluated using FlowJo (Treestar, Inc; USA). Recombination assays were performed using an established RAG2 -/- pro-B cell line *in* *vitro* recombination assay with a stable GFP reporter activated by a recombination signal sequence (RSS) [7] and endogenous repertoire diversity as previously described [29]. *In* *vitro* B cell differentiation cultures were performed following EasySep human B cell isolation kit (StemCell; USA) via indicated cytokines (Peprotech; USA), CpG (Invivogen; USA) and MegaCD40L (Enzo; USA) [30].

### Lymphocyte Repertoire Analysis

Rearranged *TCRB* and *IGH* loci analysis was initially performed (immunoSEQ, Adaptive Biotechnologies, Washington, USA) as reported previously [31, 32]. Bulk T cell beta chain and B cell heavy chain receptor sequences and sorted subsets of B cell heavy chain receptors were performed as previously described [33]. Repertoire analysis was performed using in-house bioinformatics available in online supplemental methods.

### Protein Autoantigen Array

Autoantigen microarrays were performed at the UT Southwestern Medical Center Microarry Core Facility, Dallas, TX [34].

### Data Availability

NGS data is deposited at the NCBI sequence read archive (SRA) study accession and available upon request. All primary code is available online (https://github.com/ejallens/rag2paper).

## Results

### Clinical and Genetic Features of Patients with Hypomorphic RAG2 Deficiency

**Fig. 1:**
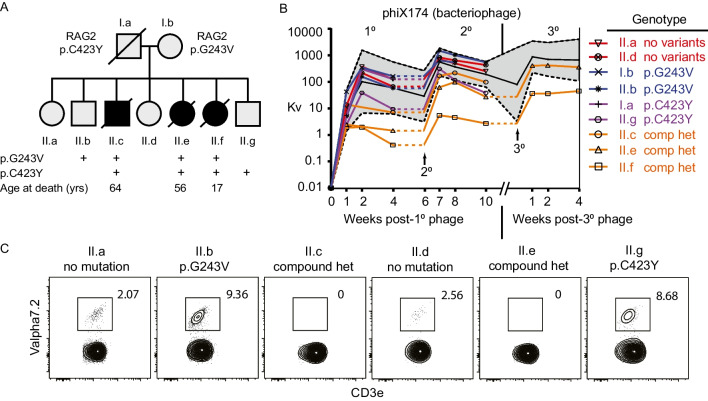
Longitudinal study of familial hypomorphic RAG2 deficiency. **(A)** Pedigree with 3 of 7 siblings with compound heterozygous variants in RAG2. **(B)** Antibody response following bacteriophage immunization demonstrates poor antibody responses via log scale titer (Kv) for family members compared to pooled reference range of adult and pediatric controls (*shaded gray*)(dashed lines indicate ± 2 SD in healthy controls; n = 58 for primary/secondary; n = 18 for tertiary). Titers were measured pre- and then 1, 2 and 4 weeks following primary, secondary or tertiary bacteriophage challenge. Siblings ranged from 18- (II.a) to 4-years (II.g) and parents (I.a and I.b) were 37- and 35-years at the time of primary challenge. **(C)** Detection of Vα7.2 segment in CD3+ T lymphocytes by flow cytometry shows that patients with compound heterozygous RAG2 variants had absence of TCR Vα7.2 expressing CD3+ T cells compared to healthy family members (II.a, II.b, II.d, II.g). Representative flow plots from thawed PMBCs gated on CD19-CD3+ cells

Here we present a detailed longitudinal study of multiple siblings affected with hypomorphic RAG deficiency **(**Fig. 1A**)**. Three siblings (II.c, II.e, II.f) eventually shared clinical features of lymphopenia, invasive bacterial and viral infections, yet lacked autoimmunity, autoimmune cytopenias (no anemia, thrombocytopenia, or neutropenia), vasculitis, or granulomas (detailed clinical summary is available in ***Supplementary Material; ***Table S1-S2). One of the affected siblings (II.f) died from sepsis during a lobar pneumonia at age 17 years, while the other two affected siblings (II.c, and II.e) lived into their fifth decades with a diagnosis of CVID or combined immunodeficiency (CID). Initial immune profiling consisted of lymphocyte enumeration and bacteriophage ϕX174 immunization of all family members **(**Fig. 1B**)**. Antibody production was measured via plaque assay to quantify phage inactivation and clearance compared with healthy controls. In healthy individuals, the standard primary and secondary immunization results in neutralizing antibody titers detectable at least to 6 years yet with steady decay, but reliably showed amplification with each booster dose. Here, the siblings with early-onset recurrent bacterial infections and lymphopenia (II.e, II.f) demonstrated blunted primary anti-bacteriophage responses, while in II.c the primary response was borderline low. Upon secondary challenge, all affected siblings (II.c, II.e, II.f) displayed depressed one-week post-immunization titers compared to controls. Three years later, affected siblings II.e and II.f underwent a third bacteriophage challenge. Both siblings showed improved neutralization, yet the antibody response for II.f remained severely depressed. These two siblings were started on immunoglobulin replacement. Sibling II.c was not clinically symptomatic initially. Whole genome sequencing (WGS) revealed compound heterozygous variants in the *RAG2* gene (c.1268G > A; p.Cys423Tyr and c.728G > T; p.Gly243Val) shared by siblings II.c and II.e. Targeted sequencing confirmed both variants were also present in the third affected sibling (II.f) from autopsy material. While we no longer had access to peripheral blood samples from II.f, the rest of the family provided serial samples to further characterize the disease. The loss of distal Vα7.2 + receptor expression on circulating T cells is a reliable biomarker for functionally testing recombination activity [35]. PBMCs from two affected siblings (II.c, II.e) lacked detectable CD3 + Vα7.2 + cells, while healthy siblings (II.a, II.b, II.d, II.g) expressed normal percentages (2–9% Vα7.2 + of CD3 + cells in blood) **(**Fig. 1C**)**. Collectively, these findings suggested both RAG2 variants were pathogenic.

### Structural and Functional Assessment of Familial RAG2 Variants

The paternal C423Y variant is a known deleterious mutation previously reported in a patient with T^−^B^−^SCID [36]. This residue at position 423 is a highly conserved zinc-coordinating cysteine in the PHD finger of RAG2 crucial in the domain’s stability [37]. In contrast, the maternal G243V variant was absent in public databases, and while highly conserved, it remained a variant of uncertain significance. We generated 3D variant models of G243V and C423Y performing structural assessment and energetic calculations **(**Figure S1**)**. Modeling revealed the G243 residue located in a connecting loop between propeller blades of the core domain but introducing G243V had minimal effect on the RAG structure or energetics. In contrast, our data suggests the C423Y change disrupts the structure of the PHD domain limiting the correct binding to chromatin **(**Figure S1**C,D)**.

**Fig. 2:**
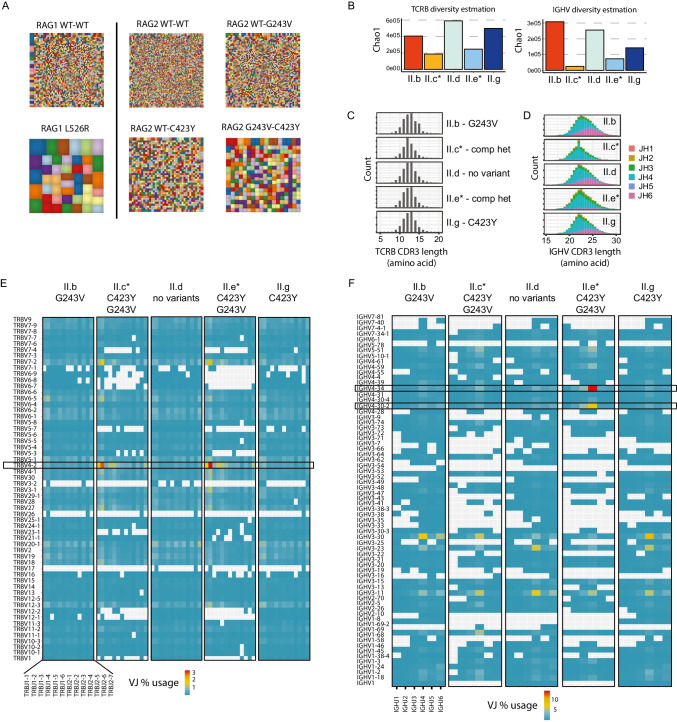
Hypomorphic RAG2 functional defect demonstrated reduced diversity and skewed VJ gene usage in siblings. **(A)** Graphical representation of the *Igh* repertoire diversity generated upon introduction of RAG1 or RAG2 variant combinations with bicistronic vectors into Rag1-/- or Rag2-/- v-Abl pro-B cells, respectively. For each bicistronic vector, the size of the color blocks correlates with the repertoire diversity. **(B)** Comparison of the antigen receptor diversity estimation for TCRB and IGHV across siblings. **(C-D)** CDR3 sequence length plotted for each J gene used per sibling comparing **(C)** TCRB or **(D)** IGH. **(E–F)** PBMC samples from each sibling were analyzed for bulk **(E)** *TCRB* or **(F)** *IGH* gene pair V and J gene usage including non-productive sequences to compare RSS sequence utilization presented as percentage of total VJ pairs per individual

To test the functional impact of the variants, we used an established *in* *vitro* recombination assay **(**Figure S2**A-D)** [7]. Introduction of the C423Y variant severely hindered recombination, while the G243V variant was comparable to WT RAG2 activity **(**Figure S2**B)**. RAG1 and RAG2 enzymes function as heterotetramers, thus we reasoned the two variants may synergistically reduce recombination. Using a bicistronic lentiviral vector, we co-expressed the variants in combinations **(**Figure S2**C,D).** Surprisingly, expression of the G243V-C423Y and WT-C423Y RAG2 combinations elicited only 18 and 19% GFP+ respectively compared to the WT-WT condition. Additionally, no significant difference was found between the WT-WT and WT-G243V combinations, both partially rescuing heterodimer function. Therefore, both the WT and G243V variants can partially rescue the recombination defect observed with the C423Y variant. Our modeling and recombination assay data suggested the G243V variant has minimal effect on recombination, thus we next assessed the potential for targeting RSS sites. The recombination cassette only contains a single recombination signal sequence (RSS) flanking the inverted GFP, whereas the murine pro-B cell line used in the assay has the endogenous mouse *Igh* repertoire with polymorphic RSS sites that can be sequenced as a second sensitive measure of recombination [7]. Thus, we compared the WT:WT bicistronic condition to the WT:G243V combination and found a reduction of nearly half as many unique reads **(**Figure S2**D).** Furthermore, the compound heterozygous condition had the fewest unique read counts. The variant effect of the two alleles could be visualized using diversity plots in combination with the WT allele or even more pronounced when expressed together **(**Fig. 2A**)**. For comparison, a known pathogenic RAG1 L526R variant from a patient with Omenn syndrome was included together with the RAG1 WT control to help illustrate the degree of recombination defect in the family reported here [38].

### Compound RAG2 Mutations Result in Restricted and Skewed TCR and BCR Repertoires

RAG deficiency typically results in an altered pre-immune repertoire with reduced *V-J* gene pairing, shorter CDR3 lengths and amino acid changes associated with autoimmunity [20, 29, 39–42]. Skewed *V-J* gene usage can partially reflect a bias for higher efficiency RSS sites [29, 39]. Therefore, genomic *TCRB* and *IGH* loci were sequenced using an unbiased multiplexed PCR technique on bulk PBMCs from affected and healthy siblings [32]. We observed a global decrease in diversity (*Chao1*) based upon CDR3 sequences in those affected (II.c and II.e) compared with their siblings (II.b, II.d, II.g) as seen with reduced *V-J* gene pairs (Fig. 2B, 2D-F) [43]. TCRB CDR3 amino acid lengths were similar amongst siblings, and we did not find increased cysteine or hydrophobic index values as previously reported for hypomorphic RAG deficiency (Fig. 2C**, S3**). When comparing across two of the affected individuals, there was a remarkably similar *TRBV-J* gene usage, especially with *TRBV04* or *TRBV07-02* paired with *TRBJ1* (Fig. 2E). We also performed immunophenotyping on PBMC samples from healthy and affected siblings by flow cytometry and apart from skewing to CD45RO + T cells as expected, we found normal percentages of both regulatory and circulating follicular helper T cells for the affected siblings (II.c and II.e) compared to a healthy sibling (Figure S3).

In contrast, the BCR repertoires were quite distinct (Fig. 2D-F). Reduced distal *JH5* and *JH6* gene usage was observed in the affected siblings as previously noted in RAG deficiency (Fig. 2D) [20, 29]. Most notably, the repertoire from sibling II.e was highly enriched for *IGHV4-34* gene usage combined with *IGHJ4* **(**Fig. 2F**).** Similar enrichment in *IGHV4-34* gene usage has been reported in several studies on the repertoire in RAG deficiency [8, 29], but the magnitude observed in sibling II.e was unique. Germline *IGHV4-34* encodes an inherently autoreactive receptor binding glycans found on blood group Ii antigens and B cells [44]. Although germline VH4-34 expressing clones account for ~ 5–10% of circulating naive B cells in healthy individuals, they are rare in germinal center and memory B cells unless somatic mutations reduce self-reactivity [45–49]. Naive cells with germline VH4-34 express anergic features with low IgM, high IgD and hyporesponsiveness [49]. The self-antigen recognition relies on a group of residues in the framework region 1 (FR1) termed the “hydrophobic patch” rather than the variable CDR3 region [45]. An idiotypic antibody, namely 9G4, binds directly to the hydrophobic patch and is highly sensitive to somatic mutations. We reasoned that the large expansion of *IGHV4-34* in the affected sibling II.e provided an opportunity to assess B cell tolerance in hypomorphic RAG deficiency using the 9G4 idiotypic antibody.

### Hypomorphic RAG Deficiency Showed Expanded CD27+ IgM+ B cells

**Fig. 3:**
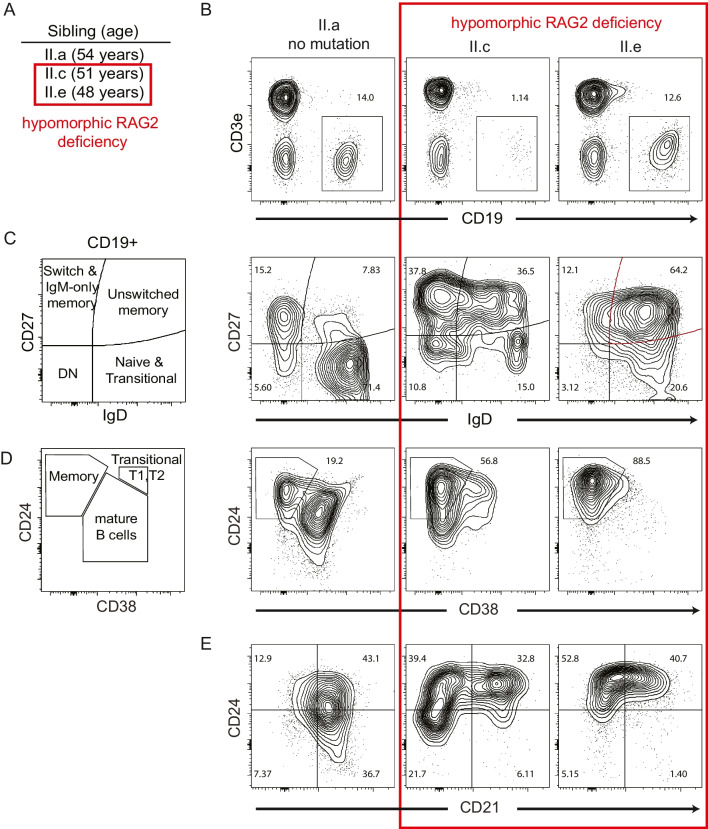
Hypomorphic RAG2 deficiency shows expanded MZ-like B cell populations and CD21^low^ B cells. **(A-E)** PBMC samples from siblings with hypomorphic RAG deficiency (II.c and II.e) compared to HC (sibling II.a) were analyzed for **(B)** total CD19+ B cells as a percentage of total lymphocytes, **(C-D)** maturation and class switching markers, and **(E)** for CD21 expression levels according to standard gating as indicated. Representative flow plots are shown

**Fig. 4:**
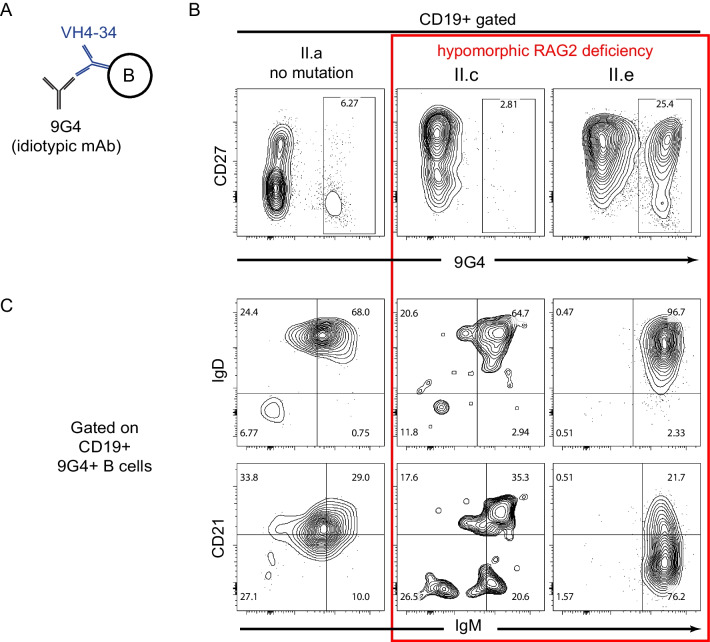
Unswitched 9G4+ MZ-like B cells are highly expanded in one sibling with RAG2 deficiency. **(A-C)** PBMC from siblings indicated in Fig. 3 were stained with an anti-idiotypic 9G4 antibody that detects a self-reactive BCR encoded by the germline *VH4-34* gene present in naive B cells and negatively selected in memory B cell populations in healthy individuals. **(B)** Idiotype 9G4+ B cells were enumerated in each sibling and **(C)** CD19+ 9G4+ cells were further immunophenotyped for maturation and class switching markers as indicated. Representative flow plots are shown

First, we performed immunophenotyping on PMBC samples from each sibling **(**Fig. 3–4**)**. Sibling II.c showed significant B cell lymphopenia, while II.e had a normal percentage of total CD19+ cells similar to their healthy sibling (II.a) **(**Fig. 3A-B**)**. Both affected individuals had an enriched percentage of unswitched memory B cells (IgM+IgD+CD27+ cells) when gated on CD19+ B cells **(**Fig. 3C-D**)**. Similar to a recent report on Tbet+ B cells in RAG deficiency, a large percentage of B cells also showed reduced CD21 expression **(**Fig. 3E**)** [20]. Sibling II.e was the most skewed withalmost exclusively high levels of IgM with a memory CD27^+^CD24^hi^ CD38^−^ phenotype and surprisingly, 25.4% of CD19 + cells stained with the 9G4 idiotypic antibody indicating expression of the self-reactive VH4-34 receptor **(**Fig. 4A-B**)**. Normal percentages of 9G4+ cells were observed in a healthy sibling (6.2%) and in the other affected sibling II.c (2.8%). Based upon longitudinal samples, the 9G4+ B cells increased over time. At age 23 years, sibling II.e showed mainly naive mature B cells (IgD^+^CD24^low^CD38^+^) with 6% 9G4 + staining (***Supplementary Material***). However, by age 47 years, a skewed population of IgD^+^CD24^hi^ cells emerged with 24% 9G4+ . Moreover, the 9G4+ B cells from II.e were uniquely showing a IgM^hi^IgD^hi^CD27^+^CD21^±^ phenotype, whereas the HC (II.a) was CD27^−^IgM^low^IgD^hi^ **(**Fig. 4C**)**. These B cells remained stable in II.e over at least a 6-year period with multiple samples tested, thus we predicted they may have other somatic mutations as she lacked any autoimmune manifestations.

### Clonally Diverse VH4-34 Found in CD27+ IgM+ B Cell Subpopulations

**Fig. 5:**
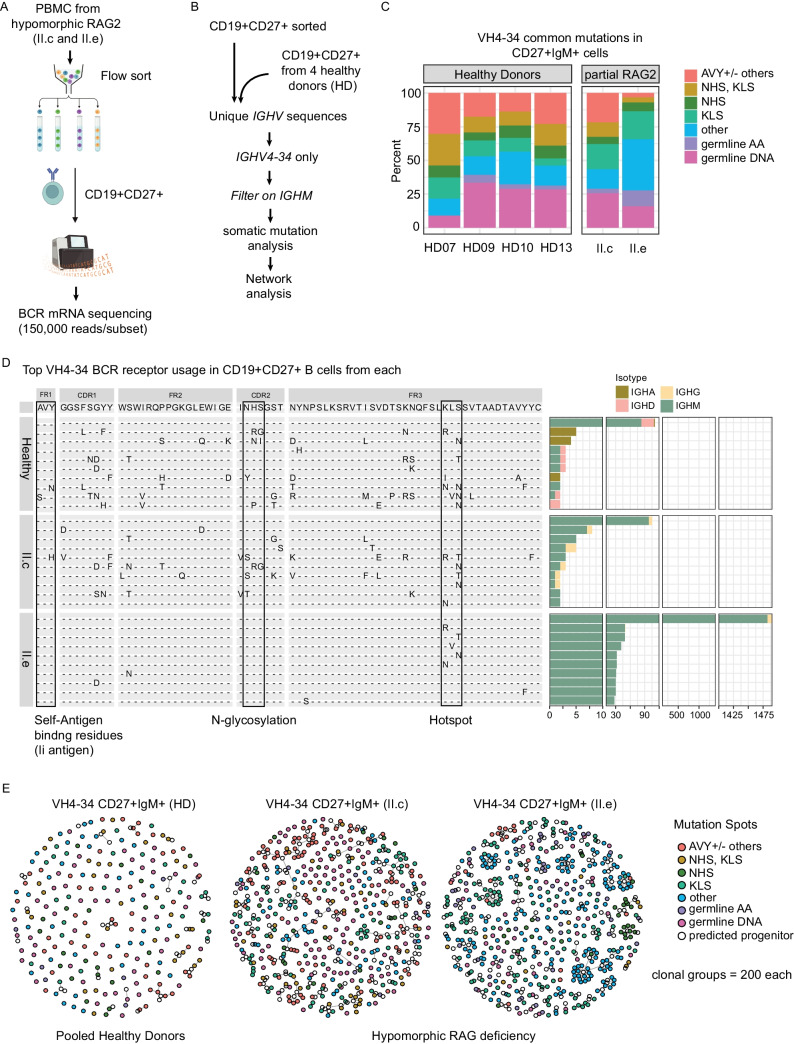
Unmutated VH4-34 receptors are enriched in CD27+ IgM+ cells in RAG2 deficiency and healthy donors. **(A-B)** PMBC from siblings with hypomorphic RAG2 deficiency (II.c and II.e) were sorted into CD19+ CD27+ memory fraction for repertoire next-generation sequencing. **(B)** Unique memory CD27+ B cell receptor heavy chain (*IGH*) mRNA sequences filtered for IGHV4-34 and *IGHM* constant region were analyzed for **(C-D)** somatic mutation analysis and **(E)** network analysis. **(C)** VH4-34 sequences were analyzed for common mutation known to reduce self-reactivity compared to germline receptors (HD07 n = 107, HD09 n = 51, HD10 n = 87, HD13 n = 72 cells). **(D)** Top 10 VH4-34 receptors with sequence shown (*left*) and number of unique B cell clones based upon CDR3 sharing this heavy chain sequence with isotypes indicated (*right*). Boxes highlight part of the hydrophobic path (AVY), N-glycosylation site (NHS) and KLS sequence as frequently mutated spots in VH4-34 to minimize autoreactivity. **(E)** Clonal connectivity plots (igraph) of VH4-34 sequences using random sampling of 200 clonal groups displayed as igraph plots somatic mutation and relatedness indicated

To characterize somatic mutations in the full length BCR heavy chain, we used transcript sequencing on sorted B cell subsets [33]. First, we sorted CD19+ CD27+ B cell populations from PBMCs isolated from affected siblings II.c and II.e **(**Fig. 5A**)**. Initially, we focused on BCR transcripts from CD27+ IgM+ B cells from the two siblings with hypomorphic RAG deficiency (II.c and II.e) and compared them to a public dataset of full length BCR sequences from sorted CD27+ IgM+ B cells from healthy donors (HD) **(**Fig. 5B**)** [50]. Transcripts were collapsed to control for any expression or amplification bias and standard immunoglobulin receptor analysis was performed to set clonal relatedness thresholds [51]. First, we focused on well described mutations in the IGHV4-34 heavy chain that can modulate antibody avidity [47, 52, 53]. Self-reactivity can be attenuated via somatic mutations in the hydrophobic patch (Q_6_W_7_ + A_24_V_25_Y_26_) in the FR1 that confers the 9G4 idiotypic antibody reactivity, the N-glycosylation site (N_57_H_58_S_59_) in the CDR2 or at residues (K_90_L_91_S_92_) in the FR3. In healthy donors, we observed ~ 30–50% of clones had receptors with AVY or NHS site mutations previously described to reduce self-reactivity (Fig. 5C**)** [47]. Surprisingly, CD27+ IgM+ cells from 3 of 4 HD demonstrated ~ 25% unmutated VH4-34 receptors at the DNA level, which was also observed in the siblings with hypomorphic RAG deficiency (II.c and II.e) suggesting this is tolerated in a portion of CD27 + IgM + B cells. Uniquely VH4-34 BCR from II.e had increased representation of isolated KLS mutations or scattered (“other”) mutations (*light green* and *blue*, respectively) across the *VH4-34* gene and also had increased silent nucleotide mutations (*purple*) in II.e compared to controls suggesting positive selection of the unmutated VH4-34 heavy chain. Conversely, AVY mutations (*pink*) in the hydrophobic patch were rare in subject II.e compared to HD samples. Although not directly tested here, we hypothesized expansion of polyclonal CD27+ IgM+ VH4-34+ B cells was secondary to an environmental exposure.

Oligoclonal expansion in B cells is common in hypomorphic RAG deficiency [20]. To test for oligoclonal expansion, we first enumerated the top 10 unique IGHV4-34 sequences from the pooled healthy donor two affected siblings and aligned by amino acid sequences **(**Fig. 5D**)**. As above, individual unique receptors were identified by *IGHV4-34* positivity with unique CDR3 sequences from sorted CD27+ B cells, but now included all isotypes. In pooled HD, a diverse population of unmutated VH4-34 receptors, each with a unique CDR3, were observed mostly of IgM or IgD isotypes (IgM = 83, IgD = 26, IgA = 2). Similarly, sibling II.c also demonstrated a diverse population of unmutated VH4-34 BCR mostly of IgM+ isotype, whereas higher AVY, NHS and KLS mutations were observed with IgG isotypes consistent with increased class switched memory B cells in sibling II.c (IgM = 98, IgG = 7). In contrast, a tenfold expansion of unmuted VH4-34 together with private CDR3 sequences were observed that were mainly IgM isotype (IgM = 1482, IgG = 7, IgA = 1). Notably, only KLS hotspot or scattered “other” mutations were observed in the top 10 VH4-34 receptors found in sibling II.e consistent with our previous analysis. Overall, we found significant numbers of diverse B cells with unmutated VH4-34 receptors in the CD27+ IgM+ population in both HD and subjects with hypomorphic RAG deficiency yet suspect an antigen-driven expansion in II.e occurred while preserving the 9G4 staining.

Next, we grouped receptors based upon the junction region for clonal relatedness [51]. We produced clonal connectivity plots from VH4-34+CD27+ IgM+ cell sequences for visualization comparing HD to the siblings with hypomorphic RAG deficiency (II.c and II.e) **(**Fig. 5E**)**. Oligoclonal expansions were observed in II.e compared to the pooled HD samples, although not as pronounced as in previous studies in hypomorphic RAG deficiency [20]. The expansions in II.e were dominated by KLS or “other” mutations and the clusters had mostly radial projections rather than elongated expansions that can indicate antigen driven selection. Elongated expansions in HD contained AVY or NHS site mutations. Analysis of VH4-34+ CD27+ IgM+ sequences from sibling II.c showed more clonal relatedness, enriched mutated and elongated clones. In both HD and affected siblings (II.c and II.e) the CD27+ B cells with germline VH4-34 receptors were mostly unique singleton clones. Thus, in healthy controls and in hypomorphic RAG deficiency, a sizable fraction of CD27+ IgM+ cells express germline IGHV4-34 sequences. Overall, the mutational landscape between siblings II.c and II.e were clearly distinct; however, our data support an antigen-driven selection in II.e with BCR retaining the hydrophobic patch in MZ-like CD27+ IgM+ B cells.

### Hypomorphic RAG Patients have Highly Polyreactive Serum IgM

Hyper IgM-like phenotypes have been observed in RAG deficiency [12, 26]. Immunoglobulin levels measured in the siblings with hypomorphic RAG deficiency suggested increasing IgM levels over time (II.e more than II.c) **(**Figure S4**)**. RAG enzymes are not required for class switching; thus we predicted B cells from II.e would have no defects in a class switching *in* *vitro* assay **(**Figure S4**)**. Purified B cells from affected sibling II.e compared to their healthy sibling (II.b) were cultured in B cell activation media for 7 days prior to staining for intracellular IgG antibody as evidence for class switching. Input cells were uniformly IgM+ CD24+ from II.e, yet after stimulation *in* *vitro*, cytoplasmic IgG was detectable in both stimulation conditions at higher levels than observed with the HC sample **(**Figure S3**)**. The B cells from II.e showed reduced class switching to IgA compared to HC. Thus, B cells from sibling II.e demonstrated the capacity for class switch recombination.

**Fig. 6:**
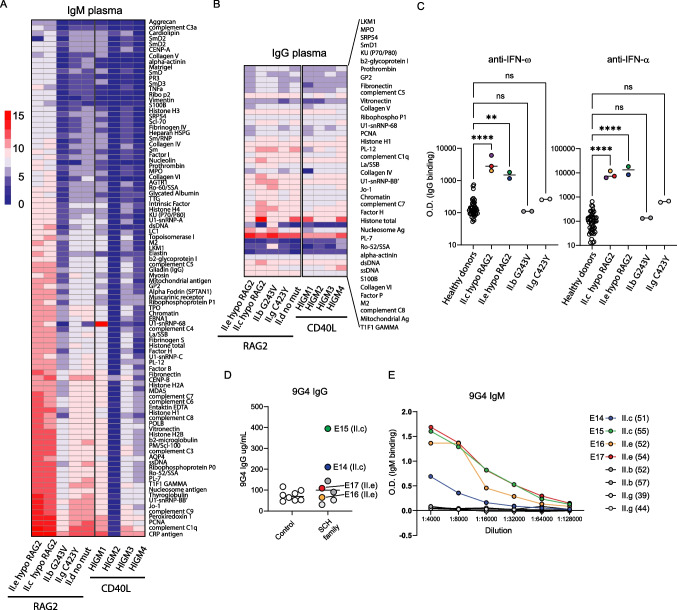
Autoantigen microarray for plasma from affected siblings compared to healthy family members and hyper IgM control samples. **(A-B)** Plasma from hypomorphic RAG2 (II.c and II.e) was analyzed by autoantigen array compared to healthy siblings or to subjects with hyperIgM (HIGM) phenotype (n = 4). Array data is normalized to the detected plasma **(A)** IgM or **(B)** IgG based upon normalized average signal. **(C)** ELISA for IgG autoantibodies to IFN-ω (*left*), IFN-α (*right*), or **(D-E)** secreted 9G4+ antibodies from hypomorphic RAG2 subjects (II.c and II.e) at two time points (E14-E17) with age indicated in parenthesis (in years). ELISA for 9G4 binding (**D**) IgG antibodies were quantified using a known standard while **(E)** IgM antibodies were compared to healthy sibling plasma samples for 9G4 reactivity using relative plasma dilution

Lastly, autoantibodies have also been associated with RAG deficiency [54]. Therefore, we measured plasma across siblings for possible autoantibodies **(**Fig. 6**)**. Plasma from healthy siblings (II.b, II.d, IIg) were compared to those with hypomorphic RAG deficiency (II.c and II.e) for autoantigen reactivity via protein microarray. We also compared plasma from several individuals diagnosed with hyper-IgM (HIGM) syndrome, both molecularly confirmed *CD40L* gene variants (HIGM1-3) or an individual with no identified gene defect (HIGM4) yet had elevated IgM levels. Notably, both siblings with hypomorphic RAG2 deficiency had elevated polyreactive IgM autoantibodies across a wide variety of autoantigens **(**Fig. 6A**)**. In contrast, the unaffected siblings and those subjects with HIGM did not show appreciable IgM self-reactivity and the IgG autoantigen array results were comparable across all samples **(**Fig. 5B**)**. Autoantibodies neutralizing type I IFN have been increasingly identified in inborn errors of immunity while uncommon in the healthy population [53]. Here we found both siblings with hypomorphic RAG deficiency (II.c and II.e) showed significantly elevated IgG autoantibodies type I IFN-α and IFN-ω across serial samples compared to both healthy controls and siblings (II.b and II.g) **(**Fig. 5C**)**. Given the expansion of 9G4+ B cells in II.e, we next measured secreted 9G4-reactive IgG and IgM antibodies in the serum of hypomorphic RAG deficient siblings (II.c and II.e) compared to healthy siblings and controls **(**Fig. 5D-E**)**. While only II.c demonstrated detectable 9G4+ IgG autoantibodies, both siblings were found to have elevated 9G4 + IgM reactivity across plasma samples. Therefore, we identified polyclonal serum specificity consistent with our skewed findings in the B cell compartments. Despite long-term clinical follow-up, the presence of self-reactive IgM was not sufficient to mediate or trigger autoimmunity.

## Discussion

Here, we demonstrate the longitudinal clinical and immunologic diversity in three affected siblings with compound heterozygous hypomorphic RAG2 variants. First, we show that compound heterozygous RAG2 variants can manifest a recombination defect only detectable by assessment in the setting of broad RSS target sequences. Previous work highlighted that RAG2 variants tested together can result in non-additive recombination activity favoring one variant [7]. Therefore, caution must be applied when labeling an isolated *RAG1* or *RAG2* variant as benign. Second, we found enrichment of IgM+IgD+CD27+ cells in two affected siblings in association with an increased frequency of CD21^low^ B cells. Similar B cell expansions were also reported in a cohort of hypomorphic RAG deficient patients [20]. Our findings expand on previous observations, showing that IgM+IgD+CD27+ cells likely represent circulating marginal zone (MZ)-like B cells enriched for weakly autoreactive specificities. The unique high expression of VH4-34 in one sibling allowed us to perform in depth analysis of B cell selection within this expanded B cell subset. Lastly, we demonstrate abundant self-reactive IgM in the serum of both affected siblings despite divergent B cell phenotypes (B cell lymphopenic versus hyper-IgM). Overall, our observations provide a unique opportunity to learn from affected siblings with identical *RAG2* variants across time. The disparate phenotypes observed across affected subjects suggests that the alterations in B cell selection observed here may be driven by environmental triggers or, more likely, reflect positive selection for self-reactivity during MZ B cell development; due to the limitations of a single-family study, this interpretation will require characterization of additional patients.

Our data collectively supports the concept that IgM+IgD+CD27+ MZ-like cells are selected in hypomorphic RAG deficiency. Broadly self-reactive IgM antibodies were present in both affected siblings. However, neither expressed clinical signs of autoimmunity and extensive clinical testing for autoantibodies (including direct antiglobulin test, cryoglobulins, antinuclear antibody, anti-thyroid antibodies and rheumatoid factor, many of which include IgM class specificities) was consistently negative. Both siblings had mesenteric lymph node biopsies revealing polyclonal kappa-skewed B cells with a splenic marginal zone lymphoma-like phenotype, without clonal populations identified. Typically, rare in circulation in healthy individuals, the frequency of IgM+IgD+CD27+ cells in sibling II.e permitted in depth characterization of such cells, demonstrating longevity, high IgM levels and largely unmutated BCRs that retained 9G4 + idiotype binding. These populations did not exhibit evidence for class switching over time, despite demonstration of the capacity to undergo class switching *in* *vitro*.

While serum 9G4+ IgM autoantibodies have been described in patients with hypomorphic RAG deficiency [20], 9G4+ B cells have not been previously characterized. Previous analysis of 9G4+ cells in healthy spleens showed a predominantly IgM+IgD+CD27+ phenotype, whereas splenic 9G4+ naive cells expressed significantly lower levels of surface IgM in healthy controls [49]. Recent work with imaging mass cytometry and single-cell RNA-sequencing clarified some of the heterogeneity in the IgM+IgD+CD27+ population including demonstration of NOTCH transcriptional signature [24, 55–57]. Two MZB IgM^hi^ subsets can be found in the blood. One subset exhibits low somatic hypermutation, preferential spatial localization in the splenic marginal zone and NOTCH pathway activation. The other subset, in contrast, is clonally related to memory B cells [56]. MZB cells are known to be enriched for self- and poly-reactivity yet are not pathogenic [25, 58]. In MZ B cells, self-reactivity does not correlate with downregulated surface IgM, unlike follicular mature B cells [59]. It is possible that these self-binding IgM antibodies are selected from antigen exposure driving polyclonal expansion and activation of polyreactive and, presumably, protective MZ-like B populations.

While a hyper-IgM phenotype has been reported in a limited subset of subjects with RAG2 deficiency, B cell immunophenotyping has not been previously reported [12, 26]. As would be expected in RAG deficiency, our patient II.e exhibited the ability to class-switch to IgG upon secondary bacteriophage challenge as well as in our *in* *vitro* studies; thus, the predominant IgM+IgD+CD27+ B cell population was surprising. We also observed normal percentages of T follicular helper cells, although it remains possible that the TCR specificity necessary to provide help to these B cell populations was discordant. Taken together, our findings support the interpretation that IgM+IgD+CD27+ B cells emerged in both siblings via a positive selection, and that this population additionally expanded in sibling II.e, presumably driven by antigen signaling. An elevated proportion of IgM+IgD+CD27+ populations, together with increased VH4-34 gene usage and elevated 9G4+ staining, has been previously reported in other primary immunodeficiency diseases including Wiskott-Aldrich Syndrome [60]. Further approaches may clarify if such populations also represent enrichment for innate MZ-like cells. In summary, we propose that IgM+IgD+CD27+ populations are likely generated via antigen driven positive selection (presumably in concert with Notch-ligand mediated survival and differentiation signals) and that an additional driver of the hyper-IgM clinical phenotype may be expansion of IgM+IgD+CD27+ populations.

In summary, we longitudinally describe 3 affected siblings with compound heterozygous hypomorphic RAG deficiency. Through detailed functional, genetic and immunological characterization we validate a novel RAG2 variant and provide evidence for positive selection and antigenic events resulting in divergent B cell immunophenotypes.

## Supplementary Information

Below is the link to the electronic supplementary material.

Supplementary file1 (PDF 864 KB)
