# Supplementary material for: Hypomorphic RAG2 Deficiency Promotes Selection of Self-Reactive B Cells
Source: J Clin Immunol. 2025 Jan 15;45(1):66. doi: 10.1007/s10875-024-01849-9 (PMC11735530; doi:10.1007/s10875-024-01849-9)
Supplement: Supplementary file 1 — Supplementary file1 (PDF 864 KB) [file 10875_2024_1849_MOESM1_ESM.pdf]

Supplemental Material

<u>Expanded Clinical Case Summaries: (</u><u>see</u> **<u>Supplemental Table S1</u>** <u>and</u> **<u>S2</u>** <u>for summarized labs</u>**<u>)</u>**

The index case (**II.f**), born in 1964, developed recurrent episodes of upper respiratory infections and otitis media at age 4 months with purulent drainage. During the second year of life, she presented with recurrent pneumonia requiring antibiotic therapy. At age 3 years, she developed extensive bronchiectasis, necessitating RLL lobectomy. Cirrhosis of the liver was diagnosed suspected from viral hepatitis with rapidly progressing jaundice, severe pruritus and transaminitis. An episode of Streptococcal septicemia and pneumococcal meningitis occurred at age 4. Due to persistent splenomegaly, she underwent splenectomy at age 8 years and due to extrahepatic biliary stenosis underwent a portocaval shunt. Skin tests for DTH (mumps, moniliasis and PPD) were negative. Schick test remained positive despite repeated immunization with diphtheria toxoid (3 times), she failed to respond to repeated typhoid vaccines, she had low isohemagglutinins, and her antibody responses to repeated exposures to the neoantigen phage ϕX174 was quantitatively and qualitatively abnormal **(Figure 1B).** Maximal bacteriophage neutralizing titers were less than 5% of normal, lacked amplification and reduced isotype switching from IgM to IgG even in the tertiary response. She initiated IM immunoglobulin therapy at age 5 years following her bacteriophage immunization demonstrating poor bacteriophage clearance. She then switched to IVIG therapy at age 9 years. She had a normal course of measles infection, yet severe chicken pox and recurrent herpes viral skin infections together with chronic dermatitis and eventual vitiligo. From a laboratory perspective, she had persistent lymphopenia with an absolute lymphocyte count (ALC) consistently near 500 cells/mm3 per manual differential. At age 9 years, she had 5% T cells and at age 12 years she had 27% T cells (vs 70.8% controls) per e-rosettes. Rectal mucosa biopsies revealed many plasma cells by histology. She died of progressive respiratory and left heart failure at 17 years. Autopsy performed revealed bronchiectasis with purulent tracheobronchitis, severe thymic atrophy, generalized lymphadenopathy and severe liver cirrhosis.

The other affected female (**II.e**), born in 1963, developed acute respiratory infections, otitis media, Staphylococcal pneumonia and an abdominal Staphylococcal skin infection during the first year of life. During that hospitalization she had WBC 21.6 with 86% polys and 2% lymphocytes. Serum electrophoresis showed low gamma globulin fraction. Schick test was positive with a negative Moloney Test and patient showed a negative isohemaglutinin anti-B test. At 2 years, she was hospitalized with *Pseudomonas aeruginosa* stomatitis. She continued to suffer from recurrent otitis media and pneumonia, impetigo, and dermatitis. She started intramuscular immunoglobulin replacement but did not tolerate the injections. At 7 years old she contracted mumps. At age 9 years, she had IgG 425, IgM 48, IgA <5 and IgE<5 mg/dL. At the age of 11 years, she developed atopic dermatitis. Antibody response to bacteriophage ϕ174 revealed moderately depressed titers with intact amplification but reduced isotype switching from IgM to IgG **(Figure 1B)**. Her Schick test remained positive despite repeated immunizations. At age 13 she experienced herpes stomatitis and was hospitalized for *Streptococcus pneumoniae* sepsis and pneumonia. At 15 years of age, she had pneumonia, herpes simplex stomatitis and underwent an appendectomy. At age 16, chronic bronchiectasis of the RL was established by imaging. She had low antibodies (IgG 255 mg/dL, IgA 3 mg/dL, IgM 35 mg/dL) and she was started on IVIG therapy. She continued to suffer from chronic lung disease and recurrent episodes of pneumonia, requiring frequent antibiotics. She continued IVIG therapy until she became pregnant at 24 years, when she discontinued replacement. She gave birth to twin females and subsequently one of her infants became septic and died, suspected to be secondary to no maternal antibodies. The other twin was given a course of IVIG considering that the baby did not receive adequate IgG antibodies from the mother. The surviving twin was immunologically normal.

At age 39 years, she experienced unexplained weight loss with an increased IgM production and underwent immunophenotyping. At the time she had lymphopenia (ALC 500 cells/mm3) with 17.2 % CD19+ B cells, 67.2% CD3+ T cells, 15.7% NK cells (CD3-CD56+) and CD4:CD8 ratio 4.8. An abnormal B cell population was observed with kappa light chains restriction concerning for marginal zone lymphoma or oligoclonal expansion. The cells were CD19+, CD45 bright with no significant low CD11c and no expression of CD5, CD10, CD25 or CD103. Ultimately, a CT scan in 2002 demonstrated cystic bronchiectasis and ground glass opacity throughout the lungs with no significant lymphadenopathy. She was treated with antibiotics and

prednisone with improvements. Follow up testing showed WBC of 7.6k/mm3 with absolute lymphocytes reported as 2052 (CD3+ 1518, CD19+ 246, CD56+ 205). Throughout her adult life, she continued to suffer from severe chronic sinusitis, oral thrush, severe asthma exacerbations and recurrent pneumonias (*Pseudomonas aeruginosa* and pneumococcal) requiring hospitalization. She had significant dental caries. At age 41, she developed significant joint pain in her hips, legs, and fingers that improved with IVIG.

The patient (**II.e**) did not return to care until early 2012 at the age of 48. At that time, she was off IVIG for 5 months. Immunoglobulins at the time were IgG 393, IgM 304, IgA 6, IgE<1. Her lymphocyte count was normal (CD3+ 1702, CD4:8 ratio 3.2, CD19+ 451, CD56+ 175). B cell immunophenotyping demonstrated an absence of immature B cells and a majority (>75%) showed a highly mature CD27+CD38^{lo}CD24^{hi} phenotype with two-thirds IgM+IgD+ and one-third IgM+ only. She had virtually no class- switched memory B cells. BAFF-R expression was normal. She was re-started on immunoglobulin replacement with adequate trough levels in the 1000-1200 mg/dl range. Her IgM level continued to be elevated at 420 and 455 mg/dL measured in 2012. Her B cells were polyclonal, subsequently confirmed by several repertoire analyses. Autoantibody screening showed negative ANA, RF, cryoglobulins, celiac antibodies. There was no history of cytopenias, and direct antibody testing (DAT) was consistently negative. Transaminases and renal function were normal with no evidence of hematuria or proteinuria. On colorectal screening she was found to have tubular adenomas that were removed, and esophagoscopy revealed Barrett's esophagitis positive for metaplasia. Both problems were resolved following treatment. In 2013, patients' mononuclear cells responded normally to PHA and anti-CD3 stimulation compared to control cells. Despite her prophylaxis, she had chronic rhinovirus infection, chronic *Pseudomonas sp* colonization, hospitalization for *Stenotrophomonas maltophilia* pneumoniae and chronic sinus infections leading to sinus surgery with *Scedosporium apiospermum complex* growth from maxillary sinuses. At the age of 55 years, she had IgG level 1270 (on IVIG), IgM 467. She had broad screening for respiratory symptoms with *Candida glabrata* detected by PCR, but no evidence of *Aspergillus fumigatus* or *zygomycete*. Mycoplasma IgM IFA was negative. She died unexpectedly on 3/13/2019 at age 55 of an opioid overdose.

The oldest of the three affected children (**II.c**) was born in 1960. Since childhood, he had frequent URI and a few episodes of pneumonia often requiring antibiotics. He was not hospitalized until as an adult. At the age of 12 years, he was unexpectedly found to be lymphopenic, similar to his 2 affected sisters, when participating in the family research study showing an ALC of 680. He had normal immunoglobulins at the time (IgG 830, IgA 80, IgM 48). His mother, and his pediatrician, considered him not immunologically impaired at that time. Skin tests for delayed hypersensitivity were negative, including PPD, mumps and monilia antigens. Schick test was negative, indicating the presence of anti-diphtheria antibodies. He had isohemaglutinin anti-B antibodies (titer 1:32). The subject was then lost to follow up, left home and did not seek medical care consistently until later in life. The punctuated records we have include documented herpes virus isolated from a mouth lesion in 1976, mild lymphopenia with ALC ranging from 1090-1300 cells/mm3, normal IgG (1055 mg/dL) and IgM (94 mg/dL), but undetectable IgA. Lymphocyte subset analysis showed ongoing fluctuations: in 1987 his counts were normal (ALC 1613, CD3 1226, CD4 890, CD8 302, CD16 269, CD20 118); in 1995, lymphocytes including T subsets were moderately reduced (ALC 1261, CD3+ 769, CD4+ 580, CD8+ 139, CD16+ 177, CD20+ 252). In the 1990's the patient was diagnosed with Hepatitis C and signs of cirrhosis. He developed chronic sinusitis confirmed in 2001 by CT scan demonstrating extensive pansinusitis with soft tissue opacification and chronic changes in maxillary and sphenoid sinuses with eventual surgical septoplasty. In 2002, bronchiectasis and lymphadenopathy were demonstrated by CT scan at the age of 42. In 2003, his lymphocyte counts were normal (ALC 1970,CD3+ 1518, CD4+ 1129, CD8+ 328, CD16+ 205, CD19+ 246). He was diagnosed with liver cirrhosis in 2004.

In 2008, he (**II.c**) was admitted to the hospital for abdominal pain and concern for lymphoma. Upper endoscopy and colonoscopy revealed no gastric or intestinal inflammation, but ascending and sigmoid colonic polyps were histologically consistent with tubular adenomas. An abdominal lymph node biopsy showed histiocytosis with plasmacytoid/plasmacytic cells scattered amongst the lymphoid cells consisting of CD20-positive B cell follicles surrounded by CD3-positive T cells. The germinal centers possessed B cell follicles with bcl-2-positive, IgD-positive mantle zones; CD23-positive follicular dendritic cells and bcl-2-negative, bcl-6-positive, high Ki-67 germinal centers, yet no aberrant co-expression of CD5 or CD43 on B cells. There were

patchy collections of CD138+ plasma cells predominantly in interfollicular and parafollicular areas. Light chain staining showed increased kappa/lambda ratio of 4:1 to 5:1. Flow cytometric studies confirmed the B cells exhibit preferential kappa light chain expression with a kappa to lambda ratio of 6.4 with excess kappa expressing B cells comprising 16.3% of the leukocytes. No clonal IgH rearrangements were identified by PCR and testing was negative for translocation involving the IgH gene by FISH, specifically negative for t(11;18)(q21:q21) by FISH. In the absence of malignancy, he was discharged for outpatient care.

In 2012, **II.c** continued to live as a recluse, only seeking medical attention for acute problems. He developed two episodes of herpes zoster in the same year, complained of chronic cough. Bronchiectasis and prominent mediastinal lymph nodes were observed on chest CT scan. GI evaluation demonstrated Barrett's esophagus and goblet cell metaplasia without the presence of helicobacter. Plasma cells were present. Lymphocyte numbers and subsets were normal with an ALC of 2500 cells/mm3 (CD3 1703, CD4 1277, CD8 400, CD16/56 200/175, CD19 451). At the age of 59, he was admitted for chest pain, productive cough, weight loss, and bloody stool. Chest CT scan demonstrated transbronchial atypical spread of tree-in-bud opacities in right middle and both lower lobes together with a right lower lobe nodule with small effusion. Immunologic testing at that time showed IgG 1474 (not on IgRT), IgM 203, IgA <3 and IgE 3. Anti-Tetanus Toxoid antibody testing was positive at 5.66, anti-diphtheria IgG 0.1 IU/mL, and anti-pneumococcal polysaccharide antigens were positive (>1.3) for 17/22 serotypes. From 2019 to 2021, numerous complete blood counts revealed ALC average of 500 cells/mm3 (range 100-1100 cells/mm3). In 2021, late-stage liver disease associated with ascites developed. He started HCV antiviral therapy given a high viral load. He died in 2024 at age 64 years.

The parents (**I.a** and **I.b**) and siblings (**II.a, II.b, II.d, II.g**) were reportedly healthy and participated in the study at the time. In 1975, when assessed by e-rosette formation all had normal circulating lymphocytes. The father (I.a) had 67.5% T cells and mother (I.b) had 60.5% T cells (vs 73.4% controls) based upon E-rosettes in 1974-1976. One unaffected sister (II.a) at age 17 had 61% T cells (vs 75.8% controls) and unaffected brother (II.g), who carried the p.C423Y variant, at age 2.5 years had 49% T cells (vs 73.4% controls). Bacteriophage studies were performed at similar times across all family members. The parents (I.a and I.b) and siblings (II.a-II.g) were immunized with Bacteriophage ϕX174 at week 0 and blood drawn at weeks 1, 2 and 4 following immunization. All individuals received a primary (1°) dose and all except parent I.a received secondary (2°) immunizations; only symptomatic siblings, II.e and II.f, were given a tertiary (3°) dose. Class switching measured by 2-mercaptoethanol susceptibility and gel filtration following the 2° dose was intact but quantitatively reduced in patients II.e and II.f compared to healthy family members and controls.

TABLE S1. Immune characteristics of siblings with hypomorphic RAG2 variants

Pt	RAG2 variants	DOB	E-rosette & Igs	Bacteriophage 1° & 2°dose	Bacteriophage 3° dose	9G4 staining	Valpha7.2	Bulk T/B Repertoire	B cell phenotype and repertoire
I.a	p.C423Y	1938	1974-76	37 yrs					
I.b	no mutation	1940	1974-76	35 yrs					
II.a	p.G243V	1957	1974-76	18 yrs		59 yrs	54 yrs	54 yrs	59 yrs
II.b	p.G243V	1959	1974-76	16 yrs		57 yrs	52 yrs	52 yrs	57 yrs
II.c	p.C423Y;G243V	1960	1974-76	12 yrs		56 yrs	51 yrs	51 yrs	56 yrs
II.d	no mutation	1962	1974-76	13yrs		54 yrs	49 yrs	49 yrs	54 yrs
II.e	p.C423Y;G243V	1963	1974-76	9 yrs	16 yrs	23,40,47,53,54 yrs	48 yrs	48 yrs	53 yrs; 54 yrs
II.f	p.C423Y;G243V	1964	1974-76	5 yrs	8yrs				
II.g	p.C423Y	1972	1974-76	4 yrs		44 yrs	39 yrs	39 yrs	44 yrs

TABLE S2. Immune characteristics of siblings with hypomorphic RAG2 variants

Patient – II.c	II.c				
Nucleotide change	RAG2 (c.1268G>A)<br>RAG2 (c.728G>T)				
Protein Change	RAG2 (p.C423Y)<br>RAG2 (p.G243V)				
**Age**	12 years	27 years	35 years	43 years	52 years
**WBC count (10^3/uL)**	3.4 (nl 4.5-13.5)				
Neutrophils (cells/uL)	2340				
Monocytes (cells/uL)	260				
Eosinophils (cells/uL)	860				
Lymphocyte (cells/uL)	680	1613	1261	1970	2500
** E-rosettes (nl ~70%)	36%				
CD3 (absolute/uL)		1226	769	1518	1703
CD4 (absolute/uL)		890	580	1129	1277
CD8 (absolute/uL)		302	139	328	400
NK CD16/56 (absolute/uL)		269	177	205	200
CD19 (absolute/uL)		118	252	246	451
TCR-alpha-beta (%)					
**Immunoglobulins**					
IgG (mg/dL)	830				1474 (no IVIG)
IgA (mg/dL)	80				<3
IgM (mg/dL)	48				203
IgE (IU/mL)					1
**Specific Antibodies**					
Anti-tetanus (IU/mL)					5.66
Anti-diphtheria (IU/mL)					0.1
Anti-pneumococcal titers					17/23 positive

TABLE S2 (cont). Immune characteristics of siblings with hypomorphic RAG2 variants

Patient – II.e	II.e						
Nucleotide change	RAG2 (c.1268G>A)<br>RAG2 (c.728G>T)						
Protein Change	RAG2 (p.C423Y)<br>RAG2 (p.G243V)						
**Age**	14 mo	9 years	16 years	28 years	39 years	48 years	55 years
**WBC count (10^3/uL)**	21.6		10.6	16.8	7.6	9.1	
Neutrophils (cells/uL)	18600		9010		4180	5205	
Monocytes (cells/uL)	2600		480		988	946	
Eosinophils (cells/uL)			370		684	346	
Lymphocyte (cells/uL)	432		320		1520	2502	
*E-rosettes (nl ~70%)		45%					
CD3 (absolute/uL)				1226	1518	1702	1702
CD4 (absolute/uL)				890	1129	1277	1277
CD8 (absolute/uL)				302	328	400	400
NK CD16/56 (absolute/uL)				269	205	175	175
CD19 (absolute/uL)				118	246	451	451
TCR-alpha-beta (%)						98%	98%
**Immunoglobulins**							
IgG (mg/dL)		425	255*		759 (on IVIG)	393*	393*
IgA (mg/dL)		5	3			6	6
IgM (mg/dL)		48	35			304	304
IgE (IU/mL)		5				<1	<1
**Specific Antibodies**							
Anti-tetanus (IU/mL)						0.48	
Anti-diphtheria (IU/mL)						0.03	

* NOTE: these values were measured off IVIG for 5 months

TABLE S2 (cont). Immune characteristics of siblings with hypomorphic RAG2 variants

Patient – II.f	II.f		
Nucleotide change	RAG2 (c.1268G>A)<br>RAG2 (c.728G>T)		
Protein Change	RAG2 (p.C423Y)<br>RAG2 (p.G243V)		
**Age**	9 years	11 years	12 years
**WBC count (10^3/uL)**	21.0 (nl 5.0-13.5)	15.8 (nl 5.0-13.5)	9.5 (nl 5.0-13.5)
Neutrophils (cells/uL)	16700	12810	6360
Monocytes (cells/uL)	1990	1250	1520
Eosinophils (cells/uL)	630	1020	660
Lymphocyte (cells/uL)	1360	620	760
** E-rosettes (nl ~70%)	5%	27%	
CD3 (absolute/uL)			
CD4 (absolute/uL)			
CD8 (absolute/uL)			
NK CD16/56 (absolute/uL)			
CD19 (absolute/uL)			
TCR-alpha-beta (%)			
**Immunoglobulins**			
IgG (mg/dL)	1150 (on IVIG)		
IgA (mg/dL)	210		
IgM (mg/dL)	150		
IgE (IU/mL)			

A

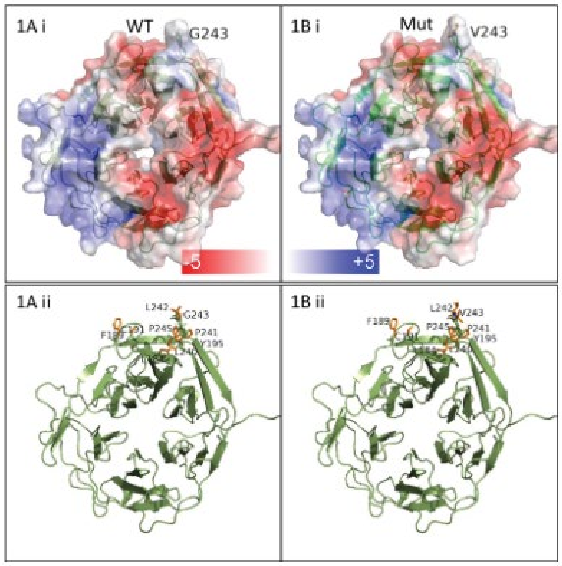

B

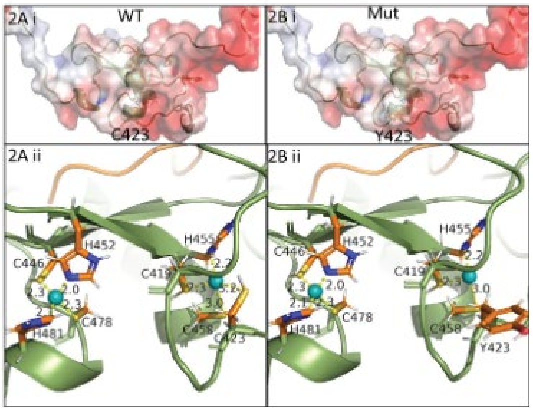

C

Variants	Total Energy	Van der Waals	Solv Hydro	Solv Polar	VdW Clash	Torsion Clash	Partial Covalent Bond
G243V	0.204	-0.218	-0.177	0.447	-0.003	0.044	0.000
C423Y	13.497	-0.559	-0.751	0.721	3.915	1.883	7.322

Note: All the energy values are in kcal/mol

D

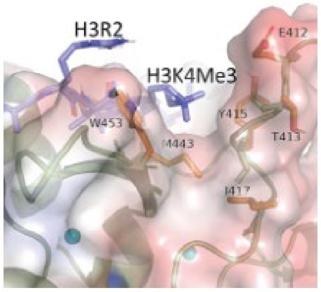

Figure S1. Modeling of the RAG2 variants demonstrates key features of mutated sites. 3D model of core domain of human RAG2 (residue 1-350 aa) was based on the mouse RAG1/2 core complex CryoEM model (PDB: 6oem; resolution 3.7Å) and C-terminal domain (PHD, residue 410-480 aa) was using a high-resolution mouse template (PDB: 2V88; resolution 2.0Å). Wildtype (WT) and mutant (mut) variants were modeled for each residue. **(A)** The core domain of RAG2 is a 6-bladed β-propeller protein with each blade of the fold connected to the next with short loops that alternatingly extend above and below the propeller and are solvent exposed. The residue of G243 is present in a connecting loop. **(B)** The PHD domain is separated from the core domain by the stretch of disordered segment called acidic hinge. The autoinhibited form of C-terminal domain is released when the PHD interacts with H3K4Me3 (trimethylated lysine 4 of histone 3). The residue C423 is part of the Zn coordinate system which contributes to the stability of the PHD domain. **(C)** Energetic changes for RAG2 genetic variants shows a tolerated ΔΔGfold value of ~0.2kcal/mol for G243V, while energetics for C423Y suggest destabilization. (D) A significant decrease in the binding affinity of the domain with trimethylated lysine is predicted due to the loss other Zn coordination bond.

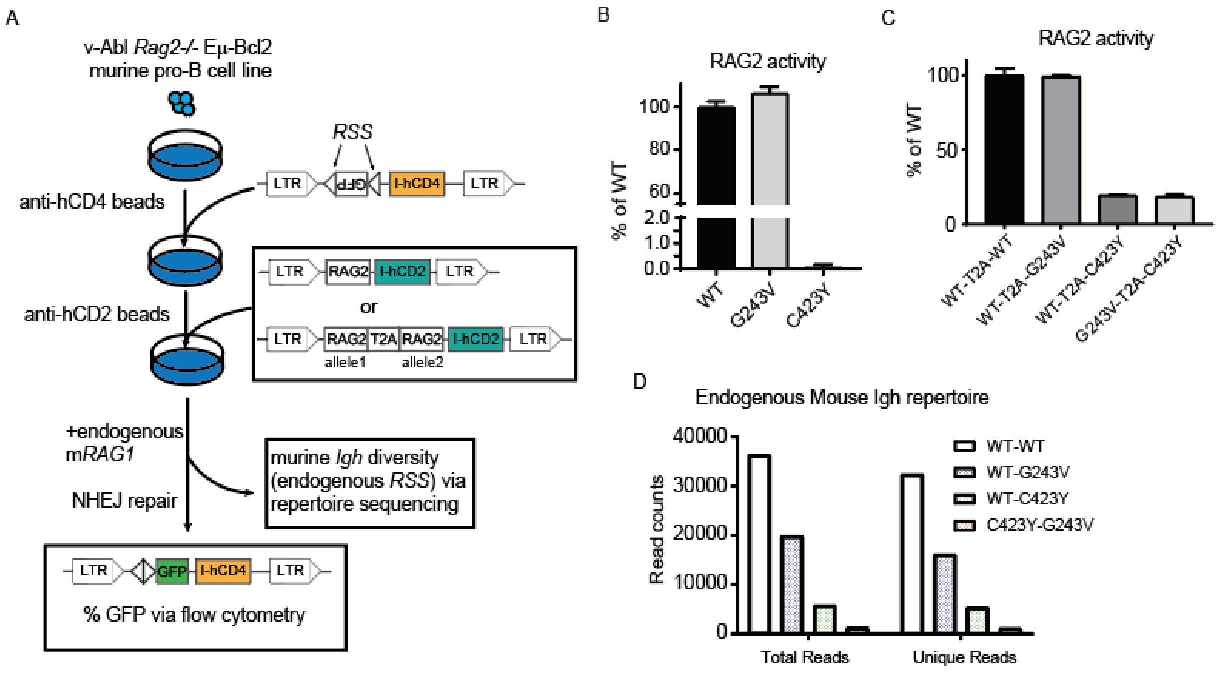

FIGURE S2. RAG2 deficiency demonstrates hypomorphic function and restricted repertoire. (A) Diagram for the *in vitro* recombination assay. The v-Abl Eμ-Bcl2 murine pro-B-cell line containing a single pMX-INV integrated inverted GFP cassette separated with IRES in cis with a human truncated hCD4 cassette was utilized and enriched for hCD4-positive cells. These cells were further transduced with a retrovirus containing either single RAG2 variants (pBMN-WT-hRAG2-IRES-hCD2) or two distinct RAG2 variants for testing compound heterozygosity using a bicistronic vector (RAG2 [allele 1]-T2A-RAG2 [allele2]-IRES-hCD2. Transduced cells with the vectors above were incubated with STI-571/imatinib to maintain cells in G0-G1 phase in the cell cycle thus allowing RAG1 and RAG2 to undergo efficient VDJ recombination resulting in GFP expression. **(B-D)** Recombination activity was assessed after 96-hours. **(B-C)** GFP expression was measured by flow cytometry gating on hCD4 and hCD2 and relative recombination activity was calculated as a percentage of WT-hRAG2 activity. **(D)** Endogenous murine *Igh* repertoire diversity was enumerated via next-generation sequencing as a surrogate readout for polymorphic RSS site utilization from the cells transduced with the bicistronic vector above.

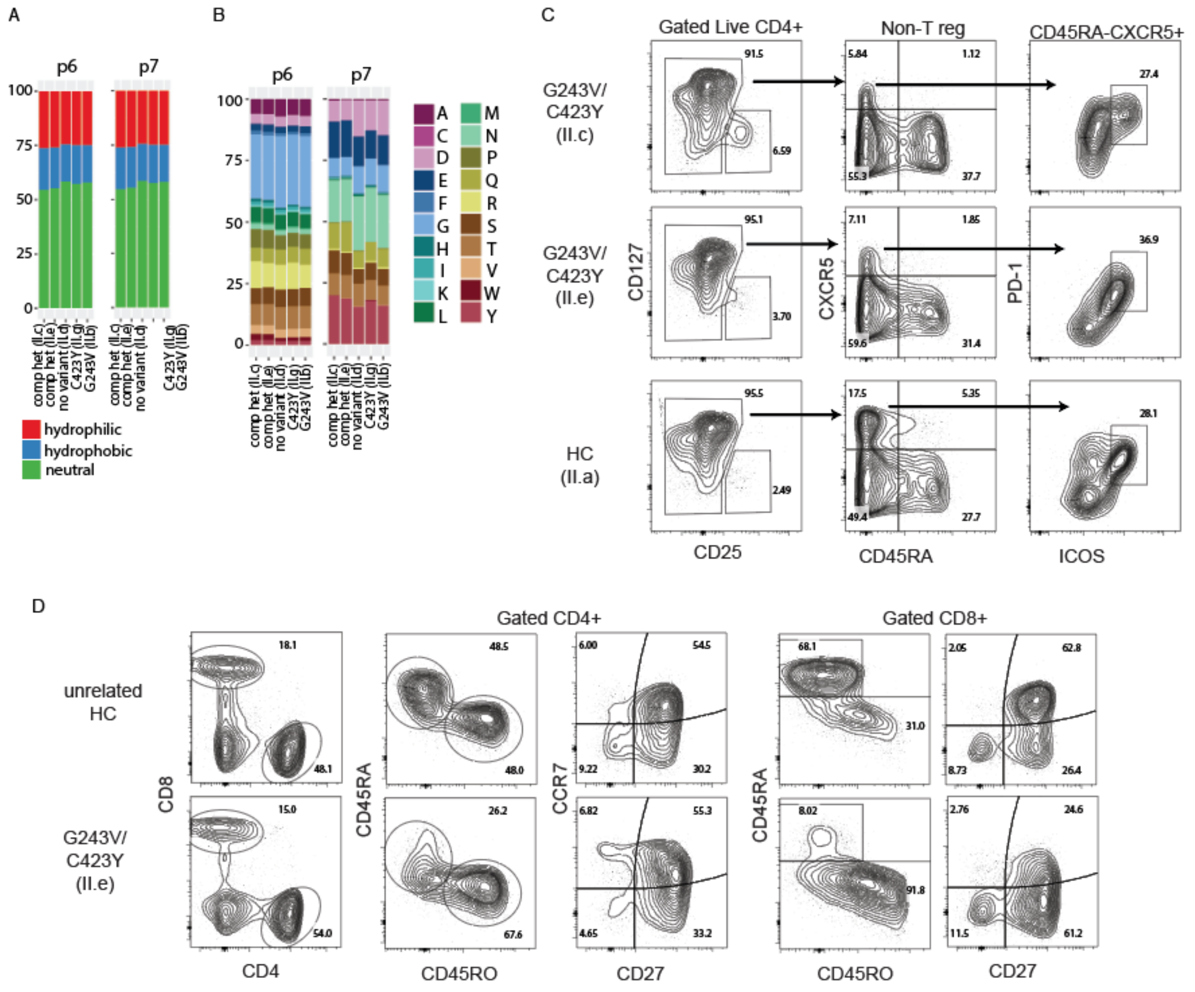

FIGURE S3. Peripheral T cells from siblings with hypomorphic RAG2 showed presence of regulatory and follicular helper T cells. (A-B) TCRB CDR3 analysis of siblings II.b, II.c, II.d, II.e and II.f for (A) hydrophobicity or (B) amino acid identity at positions p6 and p7 based upon unbiased multiplex PCR genomic sequencing data. (C) PBMC were stained for regulatory T cells (CD25+CD127low) and circulating follicular helper T cell phenotype (CD45RA-CXCR5+) in affected (II.c and II.e) and healthy control (HC) sibling (II.a). (D) Clinical T cell immunophenotyping performed for sibling II.e (age 47) from PBMC compared to healthy control.

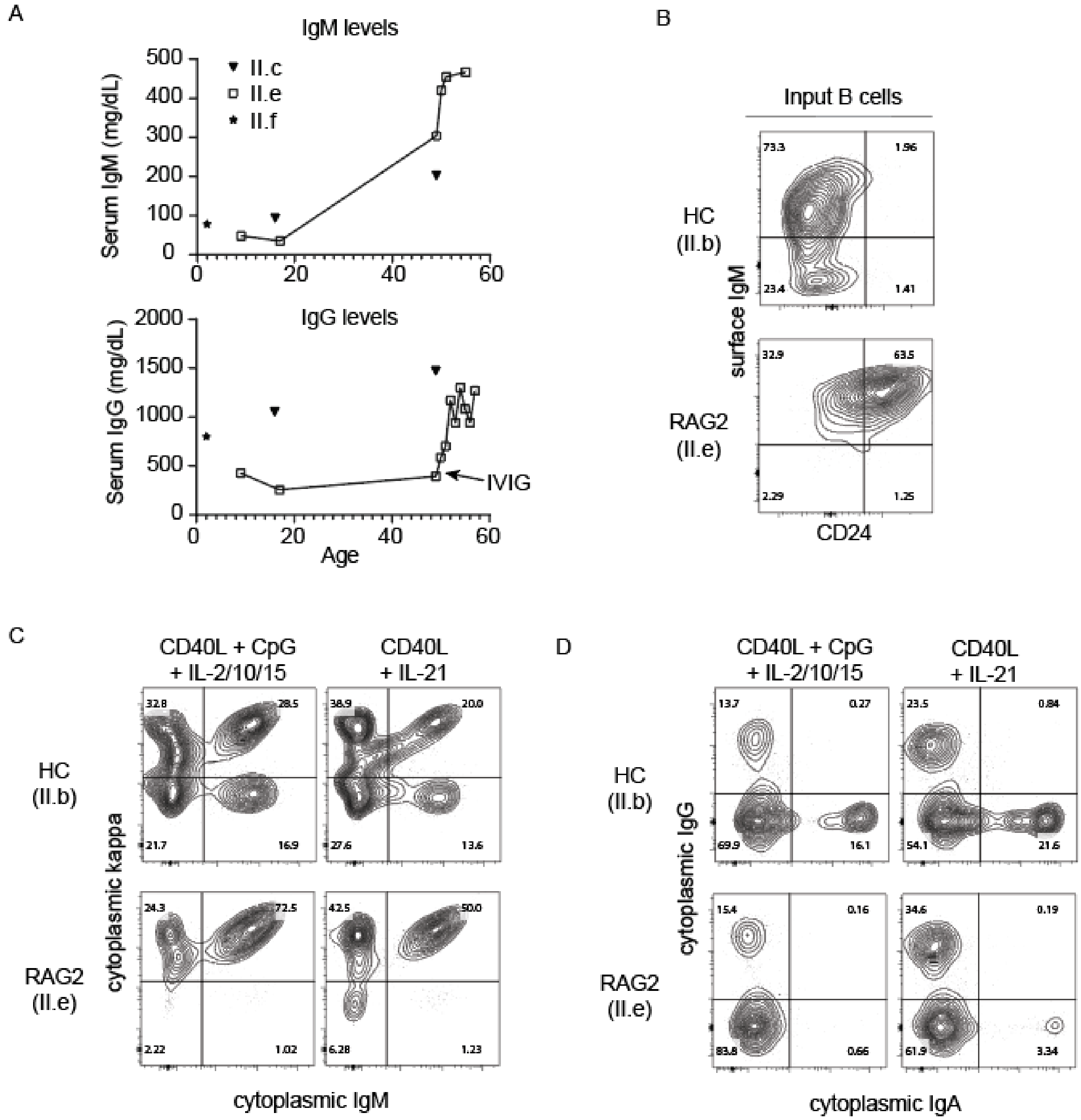

FIGURE S4. Hypomorphic RAG2 patients have increasing IgM levels yet ability to class switch. (A) Clinical immunoglobulin levels for siblings with hypomorphic RAG deficiency including IgM (top) and IgG (bottom) from the medical chart [II.c *(triangle*), II.e (*square*), and II.f (*asterisk*)]. **(B-D)** Frozen PBMC were thawed, purified and surface stained for input purity by flow cytometry as shown with sorting gating as shown **(B)** comparing a RAG2 hypomorphic subject (II.e) with hyper IgM phenotype to healthy sibling (II.b). **(C-D)** Cells were immediately cultured in B cell activation medium for seven days in 100ng/mL mega-CD40L together with either combination of either CpG (1μg/mL) and IL-2, IL-10 and IL-15 (50ng/mL each) or together with IL-21 (40ng/mL) as indicated. Cells were then harvested and stained for cell surface markers followed by intracellular Ab staining for cytoplasmic heavy and light chain.

Supplemental Methods

Immunophenotyping

Peripheral venous blood was collected in either untreated (for antibody and immunoglobulin determinations) or sodium-heparin (for genetic analysis and cell function tests) containing tubes from patients, family members and healthy adult blood donors that served as controls. All patients were treated at the Immunology Outpatient Clinic in Seattle. PBMC were used to analyze lymphocyte subpopulations by multicolor flow cytometry using standard protocols. Cells were first incubated in Human FcBlock and AF350 viability dye prior to staining with monoclonal fluorophore conjugated antibodies:

Name	Clone	dilution	Company	Cat#
CD19 anti-Human PE-Cy7	HIB19	1:50	Biolegend	302216
Anti-TCR V alpha7.2 Mouse Monoclonal Antibody APC	3C10	1:100	Biolegend	351707
CD3 Monoclonal Antibody eFluor 450	OKT3	1:100	ThermoFisher	48-0037-42
Anti-human FC Receptor Binding			ThermoFisher	14-9161-73
CD38 mouse Anti-Human BV-421	HIT2	1:50	BD Horizon	562445
CD21 Anti-Human BV510	B-ly4	1:50	BD Biosciences	740165
IgD anti-Human APC-Cy7	IA6-2	1:50	Biolegend	348218
9G4 FITC (2.42mg/ml)		1:200	Sanz laboratory	n/a
CD27 Monoclonal APC	O323	1:50	ThermoFisher	17-0279-42
CD24 Mouse Anti-Human PE	ML5	1:50	BD Biosciences	555428
CD3 anti-human BV785	SK7	1:50	Biolegend	344842
IgM anti-human PE/Dazzle594	MHM-88	1:1000	Biolegend	314529
CD197 (CCR7) anti-human PE-Cy7	G043H7	1:50	Biolegend	353226
CD25 Mouse Anti-Human BV650	M-A251	1:50	BD Biosciences	563719
CD279 (PD-1) anti-human BV421	EH12.2H7	1:25	Biolegend	329920
HLA-DR Mouse Anit-human FITC	L243	1:50	BD Biosciences	347363
CD4 Mouse anti-human BV786	L200	1:50	BD Biosciences	563914
CD8 anit-human AF700	SK1	1:50	Biolegend	344724
CD278 (ICOS) anti-human/mouse/rat APC-Cy7	C398.4A	1:50	Biolegend	313530
CD185 (CXCR5_ anti-Human APC	J252D4	1:50	Biolegend	356908
CD127 (IL-7R) Mouse Anti-Human PE-CF594	HIL-7R-M21	1:50	BD Biosciences	562397
CD45RA anit human PE	HI100	1:50	Biolegend	304108

Bacteriophage Immunization

Phage was administered intravenously at a standard dose of 2×10^9 PFU/kg body weight either once (I.a), twice (all other family members), or three times (affected siblings II.e and II.f) at least 6 weeks apart. Blood samples for antibody titers were collected prior to exposure to phage and at 1, 2 and 4 weeks after each immunization [1,2]. Antibody activity was determined by a neutralizing assay and expressed as the rate of phage inactivation (Kv) as previously described. Percent of phage specific IgG antibody was assessed two weeks after secondary immunization by determining the fraction of antibody resistant to treatment with 2-Mercaptoethanol (Percent IgG observed in 50 normal controls: 48% +/- 23% SD [3].

DNA isolation, exome sequencing and HLA-Scan analysis

Genomic DNA was prepared from peripheral blood by spin column purification (QIAamp DNA Blood Mini Kit; QIAGEN, Germany) and prepared for next generation sequencing using the TruSeq PCR-free library kit (Illumina, CA). Whole genome sequencing was performed using an Illumina HiSeq 4000 instrument (Illumina, San Diego, CA, USA) with 100bp paired end reads. Analysis of whole genome sequencing data was performed in collaboration with the University of Washington, Center for Mendelian Genomics (UW-CMG). Reads were aligned to human genome build GRCh37 using BWA-MEM algorithm. Single nucleotide variants

(SNVs) and small insertions and deletions (indels) were identified using the Genome Analysis Toolkit (GATK) Unified Genotyper, version 3.2-2. The resulting multisample VCF was decomposed (VT version 0.57), annotated (VEP version 87) and filtered for rare variants (allele frequency <0.005) according to the expected *de novo* inheritance model using GEMINI. T-cell receptor repertoire can be shaped by MHC genotype [4,5]. HLAscan, an alignment-based program that takes read distribution into account to determine true allele types [6]. HLA sequences are repeated, and their reads are randomly aligned with tiled patterns captured and scored to remove the false positive reads. The provided output score represents the probability where the reads are distributed amongst tiled array patterns and a score greater than 0.1 has normal distribution. WGS data was used to predict the HLA type for family members demonstrating the two affected individuals were 9/10 haploidentical except for HLA DQB1.

Recombination analysis of RAG2 variants
To test the *in vitro* recombination efficiency of each variant separately and when expressed together we used a standard assay with a fluorescence-based recombination assay as described previously [7]. Briefly, a V(D)J substrate plasmid, containing an inverted *GFP* cassette flanked by a fixed RSS was retrovirally introduced into *Rag2−/−* transformed mouse pro-B cell lines harboring a B cell leukemia/lymphoma 2 (*Bcl2*) transgene. The pro-B cells were transduced with retrovirus expressing either wild-type or mutant RAG2 and the pro-B cells are blocked in the G0/G1 phases of the cell cycle by treatment with imatinib, and GFP expression is measured by flow cytometry as a read-out of the recombination activity of the RAG protein. A bicistronic vector was utilized to pair wild-type RAG2 separated with a T2A sequence encoding a self-cleavable sequence with each of the mutants or pairing the variants together. Genomic DNA from the transformed murine pro-B cells was isolated to analyze the recombination activity of human RAG variants at the endogenous *Igh* locus.

High-Throughput Repertoire Sequencing Analysis
An in-house developed informatics pipeline was used for analysis of sequencing data. Bulk T cell beta chain and B cell heavy chain receptor sequences from Adaptive and sorted subsets of B cell heavy chain receptors from Illumina sequencing were submitted to the international ImMunoGeneTic (IMGT) information system's HighV-QUEST for sequence annotation [8]. Results from Illumina sequencing were done on bulk PCR amplified cell subsets and therefore errors from PCR and duplicated sequences from PCR needed to be removed. To determine unique sequences, CDR3 nucleotide sequences were combined with both the preceding and succeeding adjacent 10 base pairs to make a unique barcode. Amino acid mutations were also parsed from the IMGT mutation output tables, and trimmed to remove primer induced-mutations Remaining mutations were included as a second bar code to delineate clonally expanded cells that could still share the same CDR3 bar code. For each subject, these sequences were grouped and counted based on a combination of their barcodes, VH genes and JH genes. To exclude possible PCR error, all counts of less than 2, and non-functional sequences were removed. To preclude PCR duplication, all remaining unique combined bar codes were each counted as a single sequence for further analysis. Cleaning and parsing of IMGT tables, and all counts were done with in-house R scripts. Briefly, filtering of the unique B cell receptor sequences was done for the VH4-34 gene. The amino acid sequences were trimmed to exclude the CDR3 and JH gene and trimmed amino acid sequences were then counted for each subject. The top 25 amino acid sequences for each subject were then counted within each cell subset and normalized based on total VH4-34 sequence counts within a cell subset. IMGT annotation tables for Adaptive BCR sequencing was filtered for rearranged BCR sequences. To assess RSS and RAG2 function, the first suggested V and J gene calls were used and counted for each subject. These counts were normalized based on the number of total sequences for each subject. To analyze CDR3 composition, sequences were filtered for productive, in-frame sequences and pseudogenes were removed. The IMGT junction sequence was trimmed to remove the first and last amino acid to obtain the CDR3 as designated by IMGT.

Adaptive BCR Heavy V Gene and TCR Beta V Gene Annotation Counts
IMGT's annotation of Adaptive sequencing for all subjects was combined separately for TCRs and BCRs. Each was analyzed by first filtering out any sequences that IMGT did not find a matching V gene. Filtered sequences were then counted for the number of different V genes that IMGT suggested as possible annotation matches.

These counts were then binned based on how many possible annotation matches each had. Each bin was then divided by the sum of total sequences to determine a percentage. For each V gene, filtered sequences were also counted for the total number of different V genes annotated by IMGT, and binned based on the number of matching annotations.

Lymphocyte Repertoire Analysis
Genomic DNA was extracted from aliquots of frozen total IEC, LPC and PBMC fractions as well as sorted T cell populations using the Qiagen DNeasy blood extraction Kit (Qiagen, Gaithersburg, Maryland, USA) and sequencing of rearranged TCRB and IGH loci was initially performed (immunoSEQ, Adaptive Biotechnologies, Washington, USA) as reported previously [9,10] and described in online supplemental methods. Full-length PCR based assay using the MiSeq 2x300bp paired end reads as described previously.

TCR Beta V Gene Usage Counts
Adaptive provided TCR count data was joined with IMGT's annotation of TCR sequences using the first suggested V and J gene calls. Unaligned sequences, unproductive rearranged sequences and alignments with pseudogenes were removed. For each subject, sequences were grouped on V and J gene usage combinations and counted. Unique sequence counts were then normalized based on the total sequence counts for each subject. V and J gene usage combinations were also counted using the Adaptive TCR count data instead of unique sequences. These were then normalized based on the total Adaptive TCR count data for each subject. To better understand RAG2 dynamics between subjects, the above was repeated but with unproductive and pseudogene alignments included.

Diversity Assessment in BCR and TCR CDR3s
All productive adaptive BCR and TCR data was further analyzed for species richness for each individual using a Chao1 estimator. We used the immunarch package in R [11] to perform the calculation and visualization. Adaptive template counts were used as estimates for clone counts and estimates are performed using CDR3 amino acid sequences.

TCR Beta Position 6 and 7 Analysis
Adaptive TCR count and sequence data was filtered to remove unproductive sequences. For each subject, sequences with CDR lengths of 15 were selected. Amino acid composition at positions 6 and 7 were counted and normalized for total sequences per subject. The percentage of hydrophobic amino acids at positions 6 and 7 were also determined and normalized for total sequences per subject.

BCR and TCR CDR3 Length Analysis
Adaptive BCR count data was joined with IMGT's annotation of *IGHV* sequences. All unproductive and pseudogene sequences were removed. CDR3 amino acid sequences were obtained by trimming the first and last amino acid from IMGT annotated junction sequences, and these were used to determine length. CDR3 lengths of less than 30 amino acids were included and each J gene counted for for each individual using Adaptive's template count as a weight for each sequence. Scales were freed relative to each individual on visualization. For TCR data (*TCRB*), Adaptive sequences were filtered to remove missing junction data, CDR3 amino acid sequences were taken by trimming the first and last amino acid from the junctions and these were used to count lengths. Lengths were filtered to remove those less than 5 and greater than 20 amino acids long. Adaptive template counts were used to weight sequence counts. Scales were freed relative to each individual on visualization.

VH4-34 Usage and Mutation Analysis in Healthy Control MBC and Naive Bulk Sequencing
A healthy donor dataset was obtained from PRJNA338795 [12]. We downloaded raw fastq datasets from three healthy donors' bulk b cells (SRR4026009, SRR4026014, SRR4026015, SRR4026019, SRR4026020, SRR4026043, SRR4026044) and memory b cells (SRR4026008, SRR4026010, SRR4026011, SRR4026018, SRR4026021, SRR4026041, SRR4026042). Sequences were processed using pRESTO [13]. We removed sequences with an average Phred quality score <20. Primer sequences were masked, read 2 sequences were

annotated with UMIs from read 1, and consensus sequences were built for each UMI for each read. Reads were then assembled by overlapping ends, and if absent they were aligned to a IMGT V gene reference file and missing bases stitched in with "N"s to make full-length reads. Constant regions were annotated using reference sequences. Duplicated sequences that shared the same constant regions were counted and collapsed, and sequences with missing internal bases of 20 or more were removed. Sequences with less than 2 reads before collapsing were removed. All sequences were then submitted to IMGT for alignment. Although the data contains UMIs to identify unique cDNA transcripts, it doesn't contain barcodes to differentiate sequences from individual cells. To make appropriate comparisons with our own sequencing, we processed the IMGT tables in the same manner, but also included isotype annotations for grouping. CDR3 nucleotide sequences with 10 bases preceding and succeeding the IMGT annotations were used for a clonal barcode. Mutations in the V gene were used as a second identifier. Sequences were then collapsed based on these identifiers, V gene and J gene usage, and isotype to identify unique clones. All unproductive sequences were filtered. IMGT annotations for gene usage and mutations were used for counts, and percentages were normalized based on sequence counts for each donor and isotype.

Supplemental References:

1) Ochs HD, Davis SD, Wedgwood RJ. Immunologic responses to bacteriophage ϕX 174 in immunodeficiency diseases [Internet]. Vol. 50, Journal of Clinical Investigation. 1971. p. 2559–68
2) Ching YC, Davis SD, Wedgwood RJ. Antibody studies in hypogammaglobulinemia [Internet]. Vol. 45, Journal of Clinical Investigation. 1966. p. 1593–600
3) Renner ED, Hartl D, Rylaarsdam S, Young ML, Monaco-Shawver L, Kleiner G, et al. Comèl-Netherton syndrome defined as primary immunodeficiency. J Allergy Clin Immunol. 2009 Sep;124(3):536–43.
4) Sharon E, Sibener LV, Battle A, Fraser HB, Garcia KC, Pritchard JK. Genetic variation in MHC proteins is associated with T cell receptor expression biases. Nat Genet. 2016 Sep;48(9):995–1002
5) Gao K, Chen L, Zhang Y, Zhao Y, Wan Z, Wu J, et al. Germline-Encoded TCR-MHC Contacts Promote TCR V Gene Bias in Umbilical Cord Blood T Cell Repertoire [Internet]. Vol. 10, Frontiers in Immunology. 2019.
6) Ka S, Lee S, Hong J, Cho Y, Sung J, Kim HN, et al. HLAscan: genotyping of the HLA region using next-generation sequencing data. BMC Bioinformatics. 2017 May 12;18(1):258.
7) Tirosh I, Yamazaki Y, Frugoni F, Ververs FA, Allenspach EJ, Zhang Y, et al. Recombination activity of human recombination-activating gene 2 (RAG2) mutations and correlation with clinical phenotype. J Allergy Clin Immunol. 2019 Feb;143(2):726–35.
8) Alamyar E, Duroux P, Lefranc MP, Giudicelli V. IMGT(®) tools for the nucleotide analysis of immunoglobulin (IG) and T cell receptor (TR) V-(D)-J repertoires, polymorphisms, and IG mutations: IMGT/V-QUEST and IMGT/HighV-QUEST for NGS. Methods Mol Biol. 2012;882:569–604.
9) Robins HS, Campregher PV, Srivastava SK, Wacher A, Turtle CJ, Kahsai O, et al. Comprehensive assessment of T-cell receptor β-chain diversity in αβ T cells. Blood, The Journal of the American Society of Hematology. 2009;114(19):4099–107.
10) Carlson CS, Emerson RO, Sherwood AM, Desmarais C, Chung MW, Parsons JM, et al. Using synthetic templates to design an unbiased multiplex PCR assay. Nat Commun. 2013;4:2680.
11) ImmunoMind Team. 2019. immunarch: An R Package for Painless Bioinformatics Analysis of T-Cell and B-Cell Immune Repertoires. Zenodo. http://doi.org/10.5281/zenodo.3367200
12) Vander Heiden JA *et al.,* "Dysregulation of B Cell Repertoire Formation in Myasthenia Gravis Patients Revealed through Deep Sequencing.", *J Immunol*, 2017 Feb 15;198(4):1460-1473
13) Vander Heiden JA*, Yaari G*, Uduman M, Stern JNH, O'Connor KC, Hafler DA, Vigneault F, Kleinstein SH. pRESTO: a toolkit for processing high-throughput sequencing raw reads of lymphocyte receptor repertoires. *Bioinformatics 2014; doi: 10.1093/bioinformatics/btu138*
